# Simulated training model of ureteropyelic anastomosis in laparoscopic pyeloplasty^1^

**DOI:** 10.1590/ACB351108

**Published:** 2020-12-18

**Authors:** Juliana Cynara Santos Lima, Hermano Alexandre Lima Rocha, Francisco José Cabral Mesquita, David Augusto Batista Sá Araújo, Rômulo Augusto da Silveira, Gleydson Cesar Borges

**Affiliations:** I MD, MSc, Centro Universitário Christus, Fortaleza – CE, Brazil. Conception, acquisition and analysis of data, manuscript writing, critical revision, final approval.; II PhD, Department of Global Health and Population, Harvard T. H. Chan School of Public Health, Boston, USA, and Department of Community Health, Universidade Federal do Ceará, Fortaleza – CE, Brazil. Conception, acquisition and analysis of data, statistics analysis, manuscript writing, critical revision, final approval.; III MD, MSc, Dr. José Frota Institute and Santa Casa de Misericórdia de Fortaleza – CE, Brazil. Critical revision, finalapproval.; IV MD, Department of Community Health, Universidade Federal do Ceará, Fortaleza – CE, Brazil. Critical revision, final approval.; V MD, PhD, Santa Casa de Misericórdia de Fortaleza – CE, Brazil. Critical revision, final approval.; VI MD, Centro Universitário Christus, Fortaleza – CE, Brazil. Conception, acquisition and analysis of data, manuscript writing, critical revision, final approval.

**Keywords:** Anastomosis, Surgical, Ureter, Simulation Training, Education, Medical, Laparoscopy

## Abstract

**Purpose:**

To develop a model for simulated training of ureteropyelic anastomosis in laparoscopicpyeloplasty.

**Methods:**

Longitudinal and experimental study, with 16 participants. A synthetic instrument was produced to simulate the renal pelvis and the proximal portion of the ureter positioned on a platform within laparoscopic simulators, thereby resulting in the realistic simulation of the ureteropelvic anastomosis. A step-by-step guide was also developed for the accomplishment of the ureteropelvic anastomosis training model.

**Results:**

In the evaluation of all participants’ suture training, a decrease was found in the time needed to perform the anastomosis, with a median of 17.83 min in the 1^st^ step and 14.21 min in the last one (p = 0.01). Regarding the knots, in the 1^st^ step, 5% of them were considered firm, with an evolution to 30% in the last step (p = 0.011).

**Conclusion:**

We noticed improvement in the ability to perform the ureteropelvic anastomosis by participants with no experience with it. Therefore, even unexperienced participants can improve their skills with this training. Moreover, we observed the effectiveness of the model use, confirmed by the participants’ opinion and its validation by expert surgeons.

## Introduction

Urological congenital malformations, in most cases, present as hydronephrosis, being the main cause of ureteropelvic junction (UPJ) obstruction, which is the narrowing of the ureter in its proximal part, near the renal pelvis, and can cause reduction or paralysis of urinary flow through the ureter and develop into loss of renal function. Currently, the diagnosis of UPJ stenosis often occurs in the prenatal period, with the finding of hydronephrosis in routine examinations. In children, possible clinical manifestations usually appear after one year of age. Surgery is indicated when there is significant hydronephrosis associated with loss of renal function higher than 40%. The gold-standard surgical technique is the dismembered pyeloplasty, described by Anderson-Hynes in 1949, in which the stenosed segment is excised, and the proximal ureter is sutured to the renal pelvis. Laparoscopic surgery is recommended after 2 years of age^1^. Surgery may be open (dismembered or with pelvic flaps), endoscopic, laparoscopic or robotic, and is indicated for symptomatic or asymptomatic patients with renal function loss or increased anteroposterior diameter of the renal pelvis or hydronephrosis grades III and IV^2^.

Dissection of the proximal ureter and pelvis should be performed only in the reconstruction areas. The normal ureter, distal to the narrowed area, is incised in the lateral portion. Placing a catheter inside the ureter facilitates the suturing of the renal pelvis to the ureter. This suture can be performed using simple separate or continuous stitches, always with the knots external to the urinary flow. Absorbable suture thread is used, preferably between 5–0 and 7–0 in size. A laminar drain should be positioned in the vicinity of the anastomosis and externalized through a counterincision. There are disagreements as to whether or not catheters should be used for urine drainage. When an open nephrostomy probe is used, the use of a transanastomotic catheter is recommended to maintain an open anastomosis. Another possibility is drainage with a multiperforated transanastomotic ye-splint or an internal double J type drainage catheter. The disadvantage of internal shunt is the need for a new anesthetic procedure to remove it by cystoscopy, 1 to 2 months after the pyeloplasty.

The use of simulation in the teaching of medicine has enormous potential, both as an excellent instrument for acquiring skills and as a useful means of evaluating them^3^. The simulation can be performed in cadavers, but due to the scarcity of the latter and the ethical and moral issues related to their use, these resources should not be directed to the simulation model. Animal models are the ones that are closest to operating a living patient, as they can actually simulate bleeding and complications; however, they are high-costly and are also associated with infectious, moral and ethical concerns^4^,^5^. The simulation should be started as early as possible in the curriculum of activities of medical residents and the teaching of skills should be progressive, respecting the skill level of each individual^6^. Studies show that there is a transfer of skills learned in a simulation environment to the operating rooms, allowing a reduction in the operative time in these cases^7^.

At the moment, there is no standardized training model that can be used in the teaching of advanced laparoscopic surgery to perform a ureteropyelic anastomosis as in a pyeloplasty procedure. Therefore, the development of a systematized model focused on this problem is justified, which is the objective of the present study.

## Methods

### Ethical aspects

This study respects the ethical precepts (Norms 466/12 and 510/16 for research in human beings) and does not present the possibility of damage to the physical, biological, psychic, moral, intellectual, social, cultural or spiritual dimension of the human being, at any stage of the research or resulting from it, and was approved by the Research Ethics Committee of Centro Universitário Christus (UNICHRISTUS) under number 20469019.6.0000.5049.

### Design and setting

Longitudinal and quantitative experimental study *at* the Surgical Skills Laboratory, Centro Universitário Christus.

### Participants

The participants recruited to participate in the study comprised graduate medical students being trained in minimally-invasive surgery at Centro Universitário Christus. The participating physicians included general surgery residents, surgeons who were residents in surgical specialties, or specialists. A sample of 11 students was obtained. The study also included professionals who were the preceptors of medical residency and considered to be experts, that is, to have more than 2 years of experience in the surgical subspecialty of urology, working with laparoscopy and being a medical residency preceptor. The performance of these professionals as medical residency preceptors takes place in the following hospitals: Walter Cantídio University Hospital, César Cals General Hospital, Cancer Institute of Ceará and Santa Casa de Misericórdia de Fortaleza. The expert sample consisted of 5 participants, with a total sample of 16 participants. All participants signed the Free and Informed Consent Form. During the training, no students were excluded, and all completed the training.

### Development

Step-by-step development of the ureteropyelic anastomosis training model:

1. Manufacturing of a synthetic instrument simulating a pelvis, made of thermoplastic elastomer, shaped like a hollow hemisphere, and another one simulating a ureter, shaped like a cylindrical tube (Fig. 1);
  **Figure 1:**
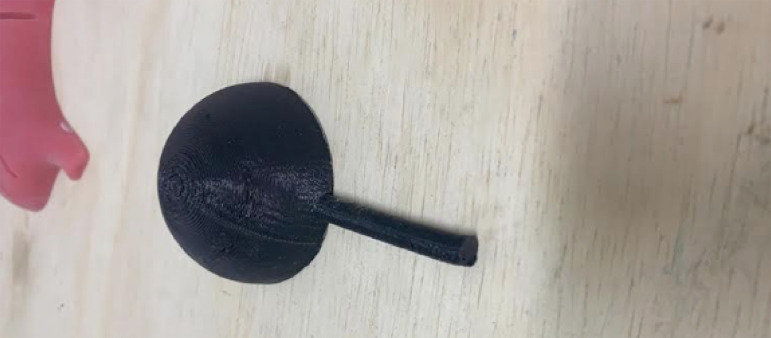
Ureteropyelic anastomosis instrument, depicting the mold creation phase for early production.
2. Fixing the instrument on an aluminum platform covered in velvety fabric, and the simulated pelvis being affixed to a circular plastic structure and a plastic screw fixing at the apex of the synthetic material semicylinder and fixed ureter with a plastic rod the platform;
3. Aluminum platform with a 45-degree angle with surface through aluminum pedestals fixed below the platform;
4. Ballast of the proximal portion of the ureter and an incision in leaf in the central area of the pelvis;
5. The position of the ureter simulator instrument and renal pelvis in a similar position *in vivo* with one material in front of the other on the platform;
6. Fixing the aluminum platform using the Endosuture Trainer Box Simulator (ESTB) (RS.ESTB-SCA-02, RS Soluções Médicas) on the right-side part at 45 degrees with it (Fig. 2);
  **Figure 2:**
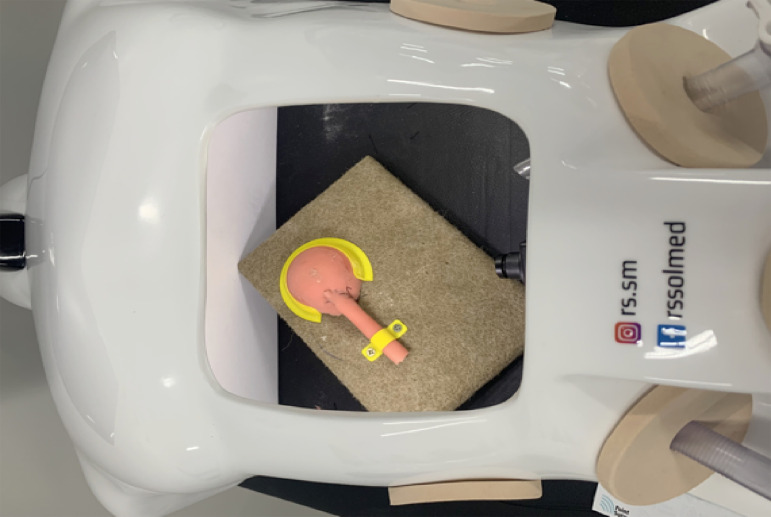
Endosuture Trainer Box(R) simulator with positioned platform.
7. Continuous suture performed with laparoscopic tweezers using 2.0 silk thread on a single plane clockwise, starting at the medial part of the anterior wall of the pelvis and in the posterior part of the tilted ureter, with initial fixation of the suture with a double knot and two single knots, as well as at the conclusion of the anastomosis.

The approval of the simulated training model of laparoscopic ureteropyelic anastomosis was carried out by five experts. They performed the preparation of anastomosis three times as the main surgeons.

The training was equally divided into three sessions and took place over a period of 4 weeks.

The training of graduate students consisted in the preparation of six anastomoses, three as the main surgeon and three as the assistant surgeon. To match the students’ schedules, the surgical team consisting of two participants was chosen by convenience.

There was no time limit for the making of the anastomosis. The end of the training was defined as the moment when the tweezers were removed from the simulators.

At the end of the students’ training, who were undergraduate students at Centro Universitário Christus, the anastomoses were compared with those of the experts and among the students themselves. These anastomoses were also compared with each other.

The participants progress using a systematized model for ureteropyelic anastomosis was evaluated. The instructors provided follow-up, performing positive feedback by stimulating and guiding participants.

The appraisal of the prepared anastomoses was carried out in two stages. First, the evaluator made a quantitative analysis using the time it took the participant to perform each anastomosis and evaluate the performance of the physicians during the training using a checklist. At a second moment, a surgeon, blinded to who has done the tasks, analyzed the final anastomoses through pictures, evaluating symmetry/regularity, asymmetry/irregularity, closure of the anterior and/or posterior wall and the presence or absence of esthesis. The firmness of the knots and the number of stitches performed to complete the anastomosis were also evaluated.

At the end of the training, all participants completed three questionnaires:

1. The first containing 10 questions about their previous experience in laparoscopic surgery and the use of laparoscopy simulators;
2. The second about their experience with ureteropyelic anastomosis and urology;
3. The third, consisting of 15 questions related to the evaluation of the proposed training model, using answers scored on the global objective structured assessment of technical skills (OSATS) assessment scale.

The global OSATS assessment scale is applied to any evaluation of surgical skills and assesses the knowledge, dexterity in the manipulation and recording of the action. It consists of seven evaluation items on a 5-point Likert scale^8^. The score for each participant ranges from a minimum of 7 points and a maximum of 35 points, and participants must obtain at least 21 points or more to be considered competent in an individual task^9^.

Both questionnaires were evaluated by surgeons not directly involved in the study. Thus, criticisms and suggestions were used to improve the instruments.

### Statistics

Categorical quantitative results were presented as percentages and counts and numerical results as measures of central tendency. Kolmogorov–Smirnov normality tests were performed for numerical variables. For categorical variables, the chi-square test was used to verify associations. For numerical measurements, the Mann–Whitney tests were used, as appropriate to the distribution of the variables. For repeated measurements, generalized linear models were used. P values < 0.05 were considered significant. The collected data were tabulated and analyzed using the SPSS software, v23, IBM, Inc.

## Results

It was observed that most of the participants were residents of the second year of general surgery (50%). The other participants comprised coloproctology residents, a digestive surgeon, urologists and another general surgeon. Among the postgraduate students, there was a nonspecialized urologist. The other participants were considered specialists and were urologists and preceptors of medical residency as predetermined in the study. Regarding the dominant hand, there was a predominance of the right-hand in 87.5% of the participants. The mean age of the participants was 34.5 years. Regarding the evaluation of the participants’ experience in laparoscopic surgery and ureteropyelic anastomosis, the surgery that was most often previously performed by the participants was laparoscopic cholecystectomy, with 100% of the participants, and the surgery with the fewest number of participants with experience was laparoscopy fundoplication, with only 12.5%.

As predetermined, none of the students had experience in laparoscopic pyeloplasty, unlike the group of specialists, in which all had previous experience.

**Figure 3:**
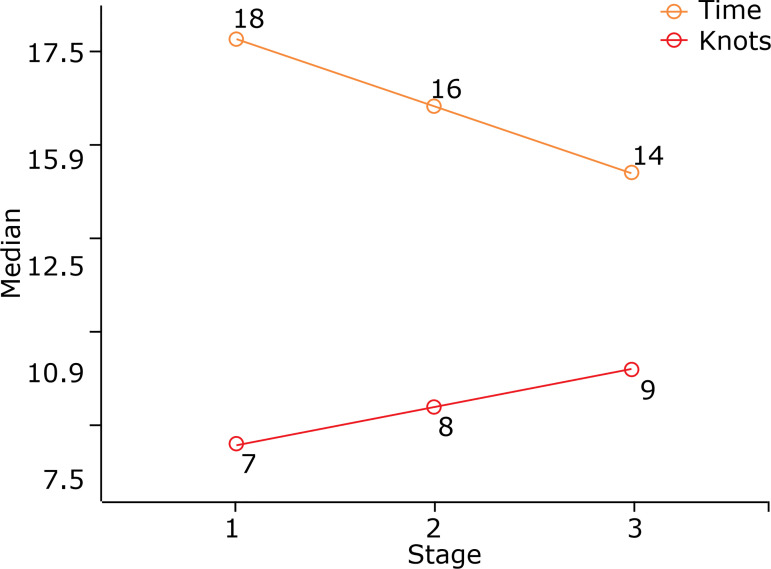
Evaluation of the evolution of participants in laparoscopic ureteropyelic anastomosis training using a systematic model described. Suture Time X Likert Scale Score (p = 0.001).

In the evaluation of the suture training of all participants, which was divided into three stages, there was a decrease in the time to perform the anastomosis, with a median of 17.83 minutes in the 1^st^ stage and 14.21 minutes in the last, with p = 0.001 (Fig. 3).

The closure of the posterior wall, which shows a greater degree of difficulty due to its position, had its closure performed by 7 participants in the 1^st^ stage and 11 in the last (p < 0.001). Comparatively, students developed with 100% effectiveness in the posterior wall closure, as well as the experts.

Regarding the parameter of firmness of the knots, it was present in 45.0% of the students and in 55.0% of the experts. Regarding the degree of symmetry, the greatest degree of symmetry was observed in the group of experts in all stages. As for the closure of the anterior wall, the two groups showed similar results. In the closure of the posterior wall, 28.6% of the students performed it, in contrast with 71.4% of the experts (p < 0.001). In the evaluation of the presence or absence of anastomosis stenosis, two students showed the presence of stenosis during the stages, unlike the experts, who showed no stenosis in any of the stages.

**Figure 4:**
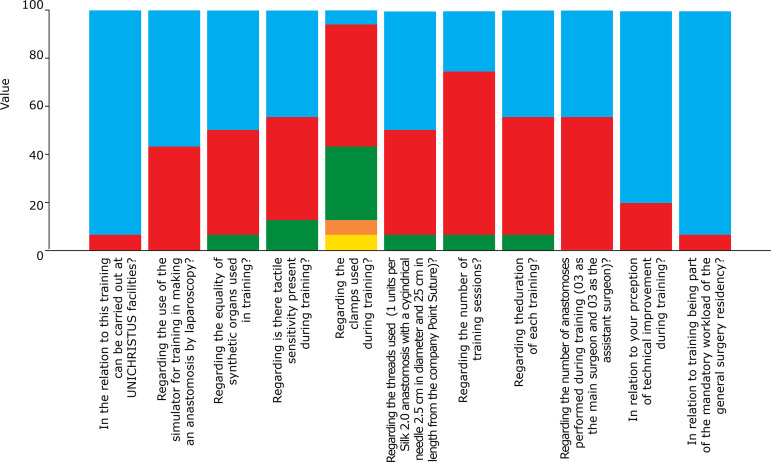
Evaluation of the laparoscopic ureteropyelic anastomosis training model.

Figure 4 shows the evaluation of the training model of laparoscopic ureteropyelic anastomosis and the majority of participants (93.8%) considered the training performance to be excellent at the Centro Universitário Christus facilities. Regarding the use of the simulator for training in the preparation of laparoscopy anastomosis, most found it excellent (56.3%) and the others considered it good (43.7%). Regarding the quality of the synthetic organs used in the training, one student participant considered it poor (6.3%) and the other ones found it good (43.7%) or excellent (50.0%). Regarding the tactile sensitivity perceived during the training, two participants considered it regular (12.5%, who were students), contrasting with 43.7% who found it excellent and good in the same proportion. Regarding the tweezers used in the training, most considered it good (50.0%) and 1 participant (6.3%) found it very bad. This student, as well as another participant that considered it bad, were students, in opposition to an expert participant (6.3%) who found it excellent. The other participants (31.2%) found it regular. Regarding the suture thread used, only one participant considered it bad (6.3%) and the other ones considered it good (43.7%) and excellent (50%). Regarding the duration of each training, 6.3%, who were all students, classified it as regular, contrasting with 50.0% who considered it good and excellent (43.7%). The ESTB simulator was evaluated as good (43.8%) and excellent (56.3%) by the study participants.

## Discussion

In the present study, the evolution of the surgical technique and the final quality of anastomosis was important, with improvement in the evaluated patterns, in addition to the absence of anastomosis stenosis in the last stage. Most parameters showed a statistically significant p-value.

Heterogeneity was observed in the initial results. During the preparation of the first anastomosis, it was verified that the students showed a great variation regarding time of performance, ranging between 13.71 to 25.98 min to perform an ureteropyelic anastomosis by laparoscopy, which was similar to that performed by the experts, ranging from 3.71 to 25.98 min, showing that there were different levels of skills. However, at the end of the training, both professionals and students showed an important reduction in the operative time to perform the anastomosis and reduced this difference to 9.18 to 19.68 min in the students, thus approaching the time required by the experts to perform the anastomoses (9.48–17.45 min). When experts are being evaluated, it is important to establish a parameter to be achieved^6^,^10^. When evaluating the suture training of all participants, the first anastomosis was performed within an average of 17.83 min, while the third anastomosis took an average of 14.21 min. Thus, in the first three anastomoses, there was a rapid acquisition of skills. In laparoscopic surgeries, increased surgical time is related to increased complications, resulting in increased hospital costs^6^,^11^. In this context, it is important to highlight the need for training, since studies show that the participation of residents is related to longer surgeries^6^,^12^,^13^.

The quality of the performed anastomoses and the surgical technique used were evaluated using the OSATS scale^6^,^9^,^14^,^15^ and a checklist. The OSATS scale is used and recognized for the evaluation of laparoscopy procedures performed in the operating room and in simulation laboratories. A series of studies have shown that this tool is considered the gold standard for providing structured feedback to residents of surgical areas and surgeons in training^14^. The checklist was adapted and based on the Master’s degree thesis by Márcio Alencar Barreira, which was created to complement the evaluation and systematize training in endosutures. Three phases of motor-skills learning were described according to observations in practical sessions^16^. The first phase is related to a rapid acquisition of skills^17^. The second is the consolidation of learning^18^. Finally, in the last phase, the gain of skills is more gradual until reaching a plateau that facilitates the retention of skills^16^. Among the participants, there was an evolution in the quality of the anastomosis, with the improvement of the degree of symmetry both in graduate students and among experts, as well as in the closure of the anterior and posterior walls and in the absence of anastomosis with esthesis.

As limitations of this study, one can mention the small number of participants, which was compensated by the use of parametric tests with more power. In addition, it was not possible to directly verify the translation of the improvement identified by training into the operating room.

## Conclusions

A systematized curricular model was developed for simulated training of ureteropyelic anastomosis in laparoscopic pyeloplasty, with the construction of a realistic synthetic simulation instrument for ureteropyelic anastomosis.

After training with the model, an evolution improvement was observed in the ability to perform ureteropyelic anastomosis by the participants without experience with it, and these have attained the final performance of anastomosis similar to that of experts. It was also concluded that even experienced participants can develop their skills with the proposed training. The effectiveness of the model use was confirmed by the participants’ opinion. Future research on the translation of competencies to the operating room are suggested.
